# A Review on Nanocarrier Mediated Treatment and Management of Triple Negative Breast Cancer: A Saudi Arabian Scenario

**DOI:** 10.3389/fonc.2022.953865

**Published:** 2022-07-22

**Authors:** Insha Nahvi, Sana Belkahla, Supratim Biswas, Suparna Chakraborty

**Affiliations:** ^1^ Department of Basic Sciences, Preparatory Year Deanship, King Faisal University, Al Hofuf, Saudi Arabia; ^2^ University of Cape Town, Department of Human Biology, Cape Town, South Africa

**Keywords:** triple negative breast cancer, nanoparticles, targetability, nano carrier and bioavailability, TNBC, nanomedications, breast cancer

## Abstract

People have continued to be petrified by the devastating effects of cancer for decades and thus a pursuit for developing anticancer agents have seen an ever-increasing trend in the past few decades. Globally, breast cancer is the most common malignancy in women and the second most common cause of cancer-related deaths. In Saudi Arabia, breast cancer is the most common type of cancer among women, constituting almost 14.2% of the total cancer burden. Triple-negative breast cancer (TNBC) is a subtype of breast cancer, which is a pathologically diverse disease of higher grade characterized by the absence of the estrogen receptor (ER), the progesterone receptor (PR), and the human epidermal growth factor receptor 2 (HER2) expressions. Despite the considerable advancements achieved in the therapeutic management of cancer, TNBC remains an unbeatable challenge, which requires immediate attention as it lacks conventional targets for treatment, leading to a poor clinical prognosis. The present research goals are directed toward the development and implementation of treatment regimens with enhanced bioavailability, targetability, minimized systemic toxicity, and improved outcomes of treatment options. The present treatment and management scenario of TNBC continues to provoke oncologists as well as nanomedical scientists to develop novel and efficient nanotherapies. Lately, scientific endeavors have addressed the importance of enhanced availability and targeted cellular uptake with minimal toxicity, which are achieved by the application of nano drug-carriers. This review intends to summarize the incidence rates of TNBC patients, the importance of nanotherapeutic options for patients suffering from TNBC, the identification of promising molecular targets, and challenges associated with the development of targeted nanotherapeutics with special reference to the Saudi Arabian context.

## 1 Introduction

In 2020, an estimated 19.3 million new cancer cases and about 10.0 million cancer deaths were reported worldwide, leading to an ever-increasing bias in the development of anticancer drugs in the recent decades. Rising population density, industrialization, and other environmental factors lead to a rise in the incidence of cancer cases universally, making scientists work overtime to discover and produce effective anticancer medications with low toxicity, high selectivity, and increased efficacy (1). According to demographics, the Saudi Arabian cancer scene reveals that in 2018 alone, there were 10,518 cancer-related deaths with 24,485 new cancer cases recorded in the kingdom. The most common types of cancer that are trending in Saudi Arabia include breast cancer, colon-rectum (CRC), and prostate cancer.

Breast cancer (BC) has the highest incidence and mortality rates of all cancer types in Saudi Arabia, with rates of 14.8% (cumulative risk of 2.87%) and 8.5% (cumulative risk of 0.81%) for both sexes, respectively. Triple-negative breast cancers (TNBCs) are a subtype of breast cancer, which is an aggressive type of breast cancer that lacks the expression of progesterone, estrogen, and human growth factor receptor 2. Therefore, it lacks particular therapeutic targets, which undermines the prognosis and its treatment becomes a huge challenge. TNBC accounts for 15–20% of all breast cancers (2).

TNBCs are finding their place in the top orders as the search for a new targetable biological traits leading to a sustainable treatment regimen is a top focus at present for researchers. Paclitaxel, classified as a “plant alkaloid,” is one of the most common chemotherapeutic drugs, used as the first-line treatment for TNBC patients. One of the most common side effects of chemotherapeutic treatments includes killing of bulk cancer cells, whereas it has been documented that chemotherapeutic treatment boosts a subpopulation of cells called cancer stem cells (CSCs), which are capable of causing new tumors. CSCs are again a substantial hurdle in the path of effective cancer treatment as it has been observed that CSCs are therapy-resistant and are instrumental in cancer progression, recurrence, and metastasis. Numerous studies have shown that the concentration of CSCs in TNBC cell lines directly contribute to the aggressive nature of TNBC (3).

The different treatment options that are used to treat TNBC patients are: conventional chemotherapy, neoadjuvant chemotherapy, adjuvant chemotherapy, PARP inhibitors, immunotherapy, etc. However, because of heterogeneity and diverse clinical characteristics, special guidelines for the conventional treatment of TNBC are lacking. However, in high-risk TNBC patients, various chemotherapy regimens, including anthracycline-taxane combos, are used. Besides, in moderate to high-risk situations, alternative treatment regimens like epirubicin, 5-fluorouracil, and cyclophosphamide in association with docetaxel (DTX) or paclitaxel (PTX) are used as adjuvant chemotherapy (2, 3). Furthermore, simultaneous neoadjuvant therapies are administered, which include platinum-based drugs such as cisplatin, carboplatin, and anthracycline, which proved more effective in the case of TNBC than adjuvant therapy. All these nanoparticles (NPs), receptor targeting compounds, and antibodies have all been employed in the therapy of hormone-negative breast cancer. Several nanoscale carrier systems have been developed as drug delivery platforms for treating TNBC in recent decades. The leaky vasculature can be penetrated by nanoformulations such as liposomes, polymeric NPs, lipid-based formulations, dendrimers, metallic nanoparticles, micelles, carbon nanoparticles, nanotubes, inorganic nanoparticles, natural agent based nanocarriers, etc. Nanomedicine that has been functionalized will be more able to be used in certain areas, such as ischemic tissue, tumors, and inflammatory areas. The drug is released because of specific enzymes, redox potential, pH and temperature activation.

This review focuses on providing a perspective on TNBCs in the Saudi Arabian context. The review aims to summarize TNBC patient incidence rates, the importance of nanotherapeutic options for TNBC patients, and the challenges associated with the development of targeted nanotherapeutics, with a focus on the Saudi Arabian context.

## 2 Classification of Breast Cancer

Breast cancers are a heterogeneous group of tumors with various classifications, and these classifications determine the type of BC that is most prevalent among the population. Various classifications that are helpful are:

1. Histopathological classification: It helps in the identification of different histologic variants of breast carcinomas, like modular, mucinous, and tubular.
2. Molecular classification: It is based on gene-expression profiling with the use of DNA microarray. Such molecular classification helps in identification into different groups like luminal A, triple negative tumors, luminal B, basal, and normal like tumors, etc. (4).
3. Immunohistochemistry: This can further help with molecular subtyping and is considered the most important test for detecting tumor sensitivity to various therapies (5).

Breast cancer is ordered into 4 major subtypes based on the presence or absence of molecular markers for estrogen receptors (ER) or progesterone receptors (PR) and human epidermal growth factor 2 (*ERBB2*; formerly *HER2*).

HR+/HER2− (“Luminal A”), HR−/HER2− (“Triple Negative”), HR+/HER2+ (“Luminal B”), HR−/HER2+ (“HER2-enriched”).

**Graph 7:**
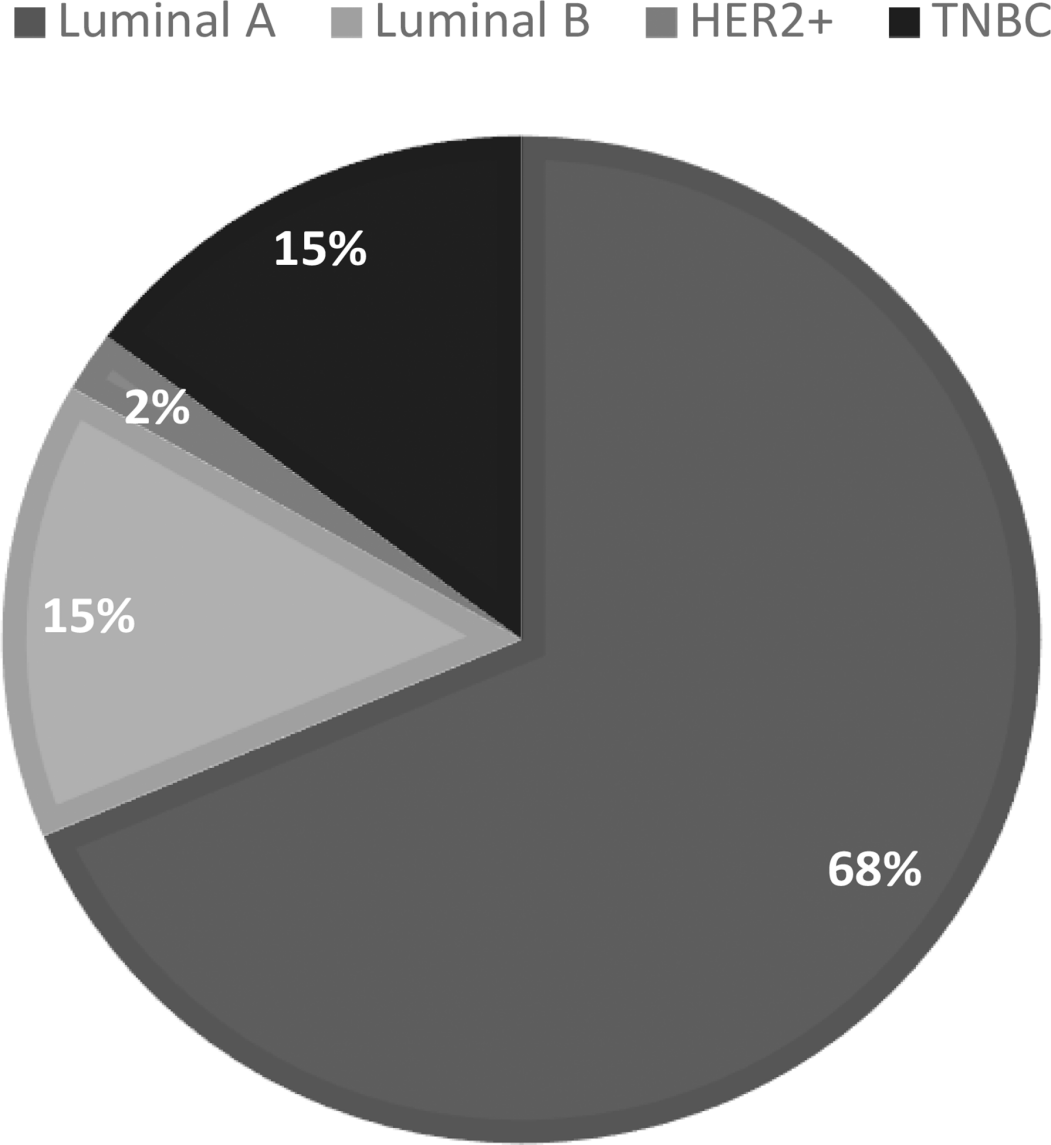
Percentage of Female Breast Cases by Cancer Subtype.

There are two ER-positive (luminal A and B) and two ER-negative intrinsic breast cancers (HER2+ and TNBC) (6) (**Graph 7**; **Table 1**).

**Table 1:** A standard immunohistochemistry (IHC) panel for the three main clinical biomarkers ER, PR, and HER2 can approximate the four intrinsic breast cancer molecular subtypes.

Breast cancer subtype	ER	PR	HER2	Triple designation	Percentage
Luminal A	+	±	−	ER+PR ± HER2−	70%
Luminal B	+	±	+	ER+PR ± HER2+	15%
HER2+	−	−	+	ER−PR−HER2+	2%
TNBC	−	−	−	ER−PR−HER2−	15%

Developed countries have a higher prevalence of BC than developing countries, and the death rate due to BC is high in developing countries. Factors responsible for the higher incidence of BC in developing countries may include dismensuria in women, obesity, hormonal therapy, extended exposure to drugs, the lack of use of vitamin supplements, indiscriminate usage of contraceptives, and having a family history of BC are crucial contributing factors (7, 8). Also, this risk continues to increase if it is the first degree relative who had BC. To identify the cause of genetic and complex diseases, genetic analysis is one of the major milestones that correlates genotype and phenotype of complex diseases like BC, as the causative variants may be identified with this plan. Saudi Arabia has successfully kicked off its first 100,000 genome project (9). This genomic revolution may help in the identification of specific genetic variants that lead to the genetic diseases among their population. This long-term project may help and contribute to disease management and better prognosis of BC, which will in fact help in establishing better genetic testing.

## 3 Breast Cancer Incidence in Saudi Arabia

Breast cancer is the leading cause of death among the Saudi population, as it is globally. In 2012, according to the Saudi Cancer Registry (SCR), 14.33% of cases of cancer were identified, with 47.5% (men) and 52.6% (women). The year 2014 marked the rise of breast cancer to the top spot among the Saudi population. A reported 15.9% of cases of BC were compared with all other cancers among Saudi nationals, and it accounts for 28.7% of all cancers among females of all ages (10). But it is noteworthy that the incidence of BC is lower but stands at second position based on the death rate due to breast cancer is 62.78% in SA when compared to other gulf countries like Bahrain, Qatar, UAE, and Kuwait (8) (**Graph 2**).

**Graph 1:**
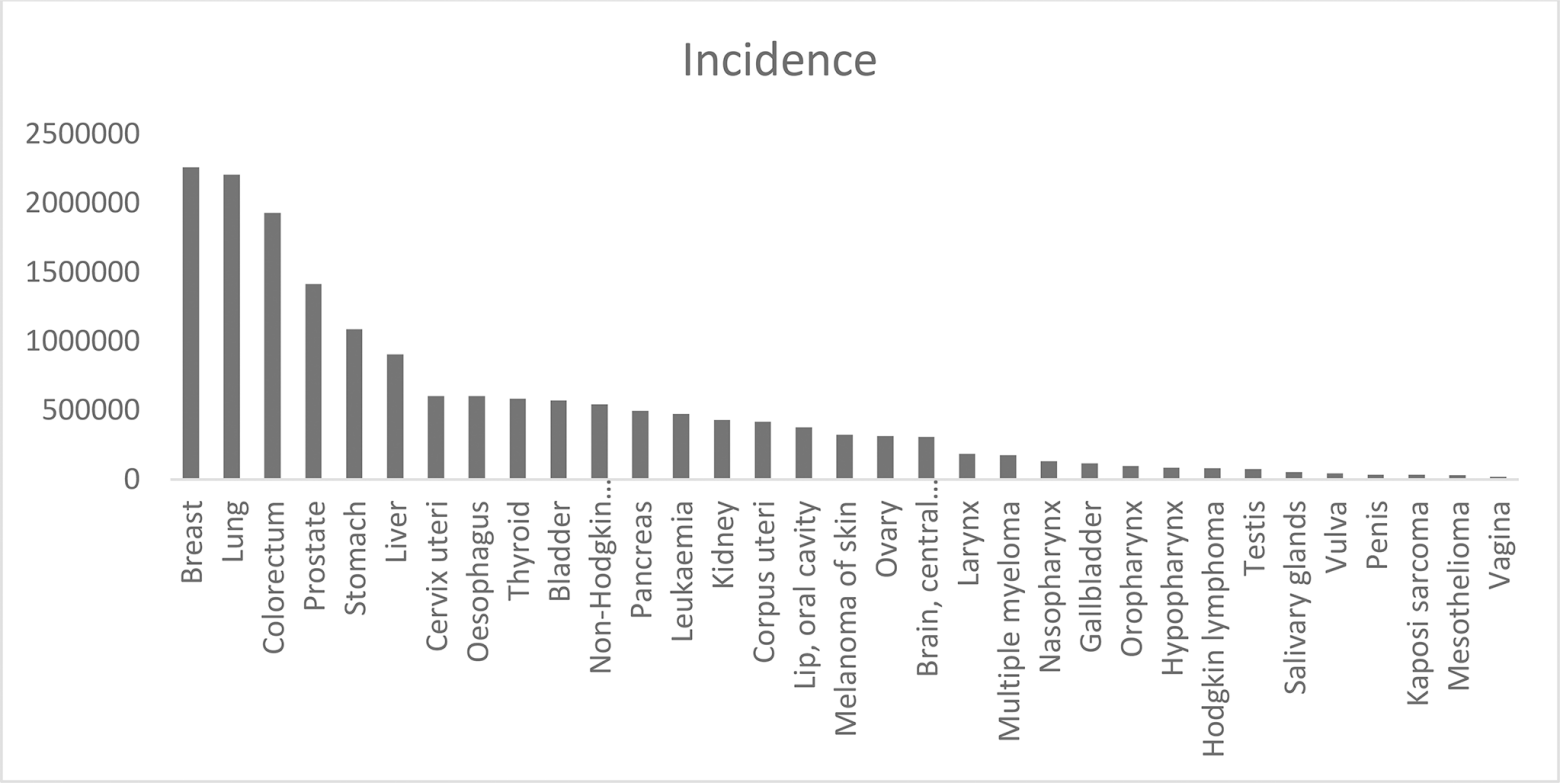
Estimated number of incident cases all cancers, both sexes, all ages 2020.

**Graph 2:**
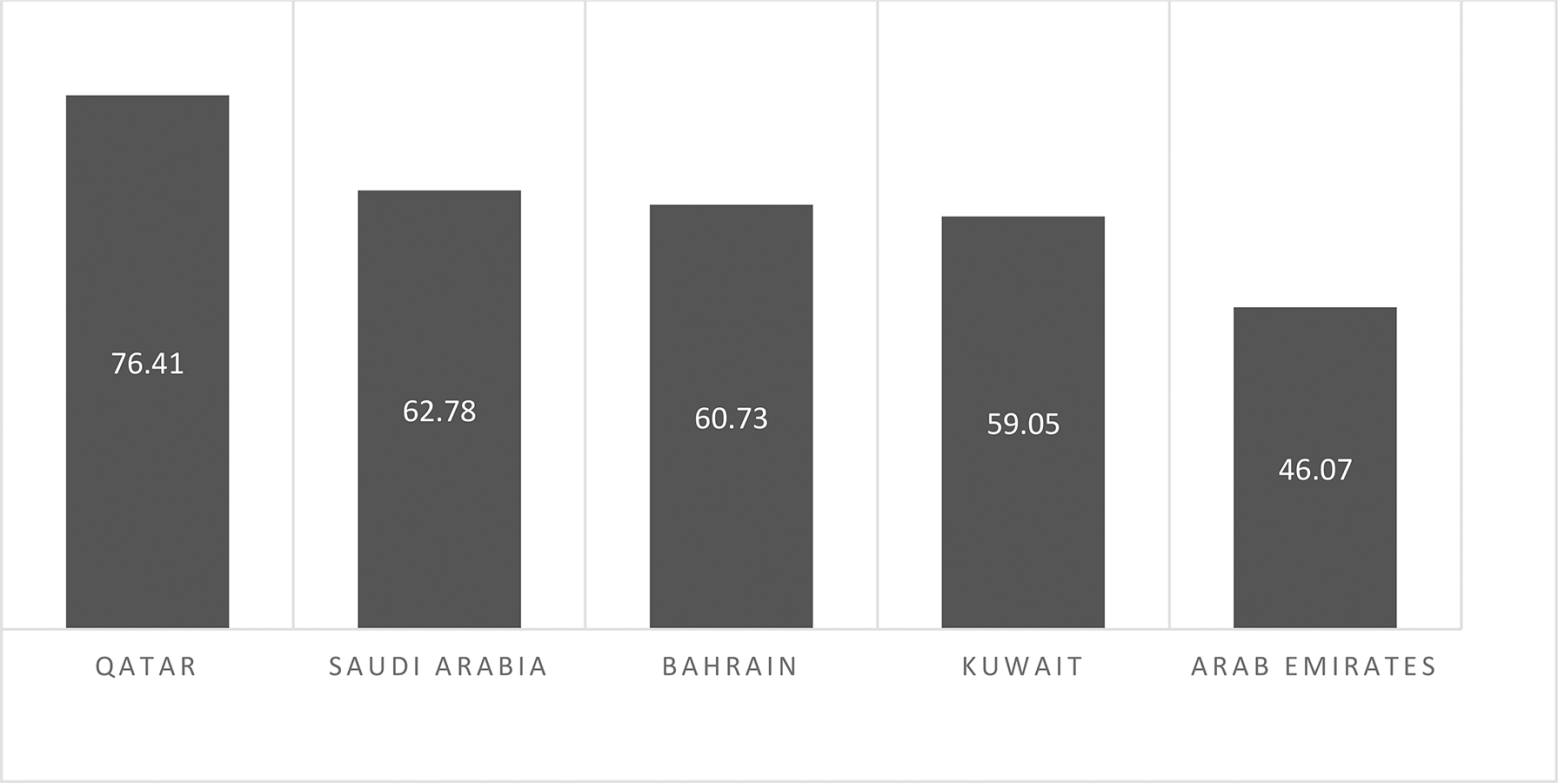
Estimated death rates/100,000 due to cancer in Saudi Arabia in comparison to other Arabian Gulf countries (WHO, 2017).

**Graph 3:**
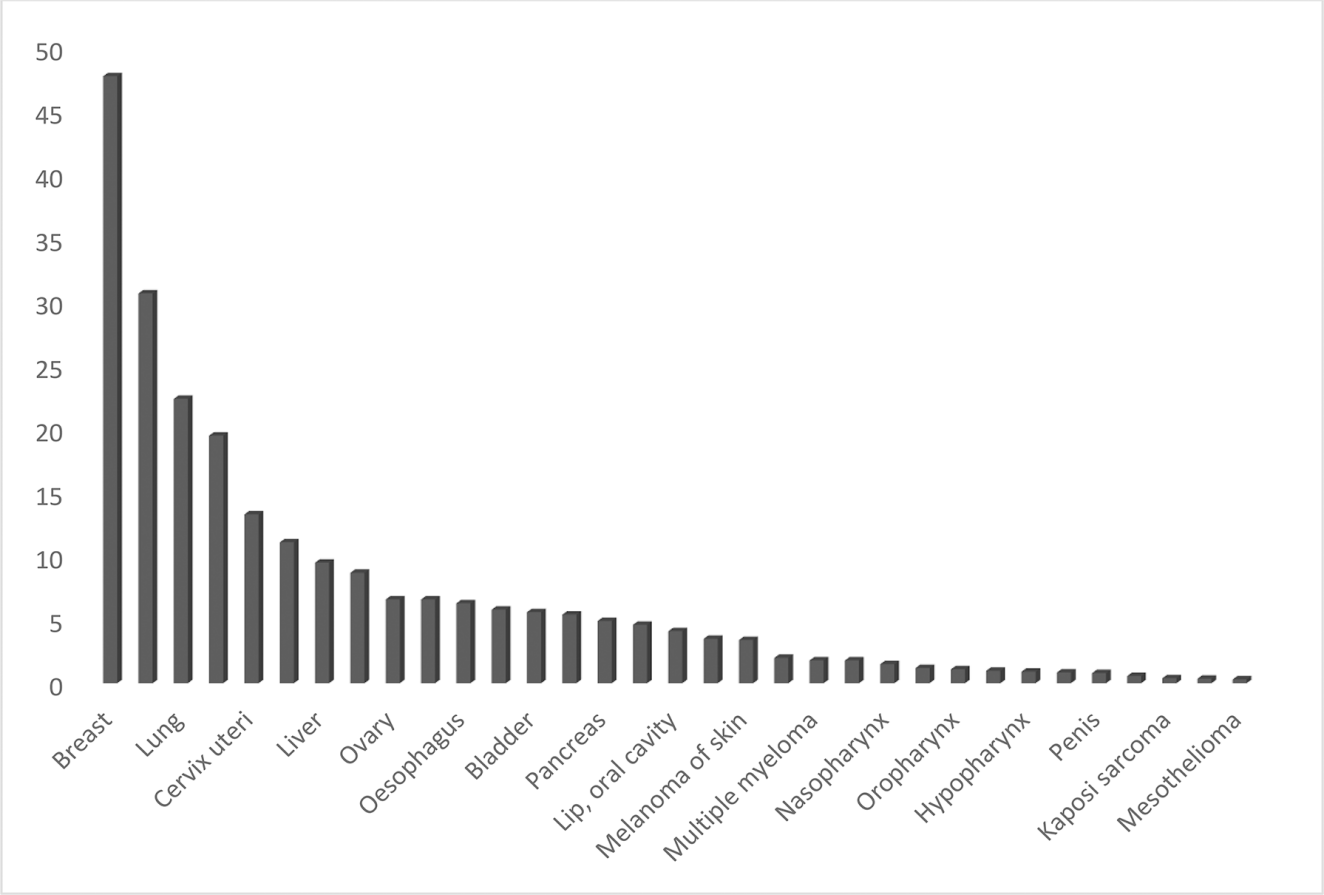
Estimated age-standardized incidence rates (World) in 2020, worldwide, both sexes, all ages.

On the basis of age-standardized rates, a study was conducted at the King Abdul Aziz University Hospital between January 2012 and December 2018 with 740 cases of breast cancer and 482 breast cancer patients admitted at the King Fahd University Hospital (KFHU) between 1998 and 2017. Around 50 years was the average age of the patients, and it was in accordance with the average age as reported by the Saudi Arabian Cancer Incidence Report (11, 12). According to these results, 54.3% of the cases were detected in women with <50 years of age, which is similar to a study from Oman, which suggested that 63.8% of cases are younger than 50 years old and 36.2% older than 50 years (13 **).** These results are also in accordance with Bahraini women. According to this study by Globocan 2020, the average age diagnosed for breast cancer was around 50 years and the median age during the 11-year period was 49 years (14) (**Graph 3**).

All these results in Middle Eastern countries were in contrast to the observations in the United States of America. According to the most recent statistical data from NCI’s Surveillance, Epidemiology and End Results (SEER) Program, women who are >55 years of age have more incidences of breast cancer (65.1%) with a median age of 62 at the time of diagnosis (15). This difference may be a consequence of a lack of high-quality healthcare systems in the Middle East compared to the US (12).

**Graph 4:**
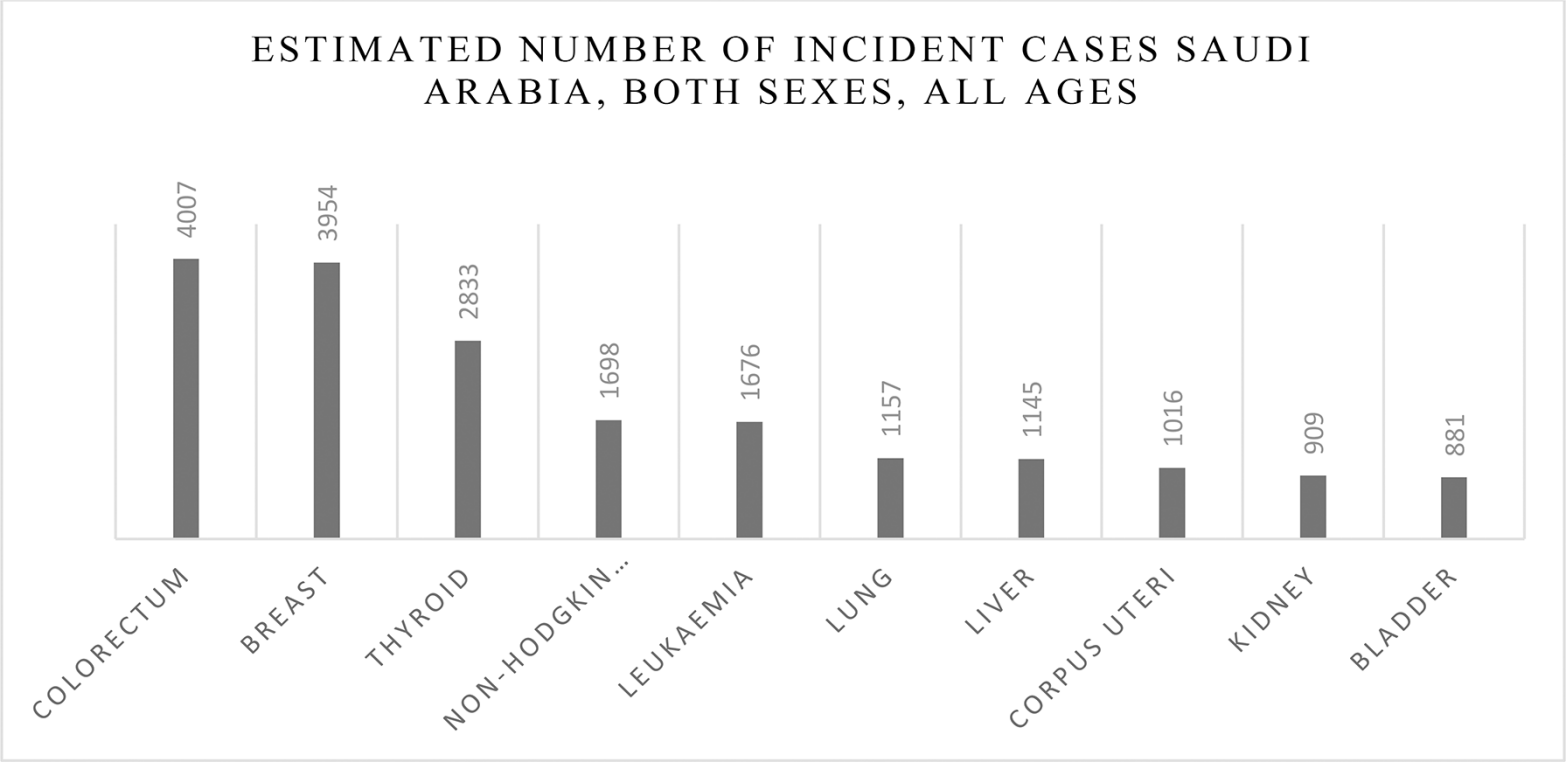
Estimated number of incident cases in Saudi Arabia, both sexes, all ages.

As per the age-standardized rates (ASR) in different types of cancers, the incidence of all cancers (5-year prevalent cases) was estimated to be 82,640 cases (39,241 men and 43,399 women) in Saudi Arabia. The mortality was 13,069 (5-year prevalent cases) cases across all ages and genders (Globocan 2020) (**Graphs 3**, **4**).

**Graph 5:**
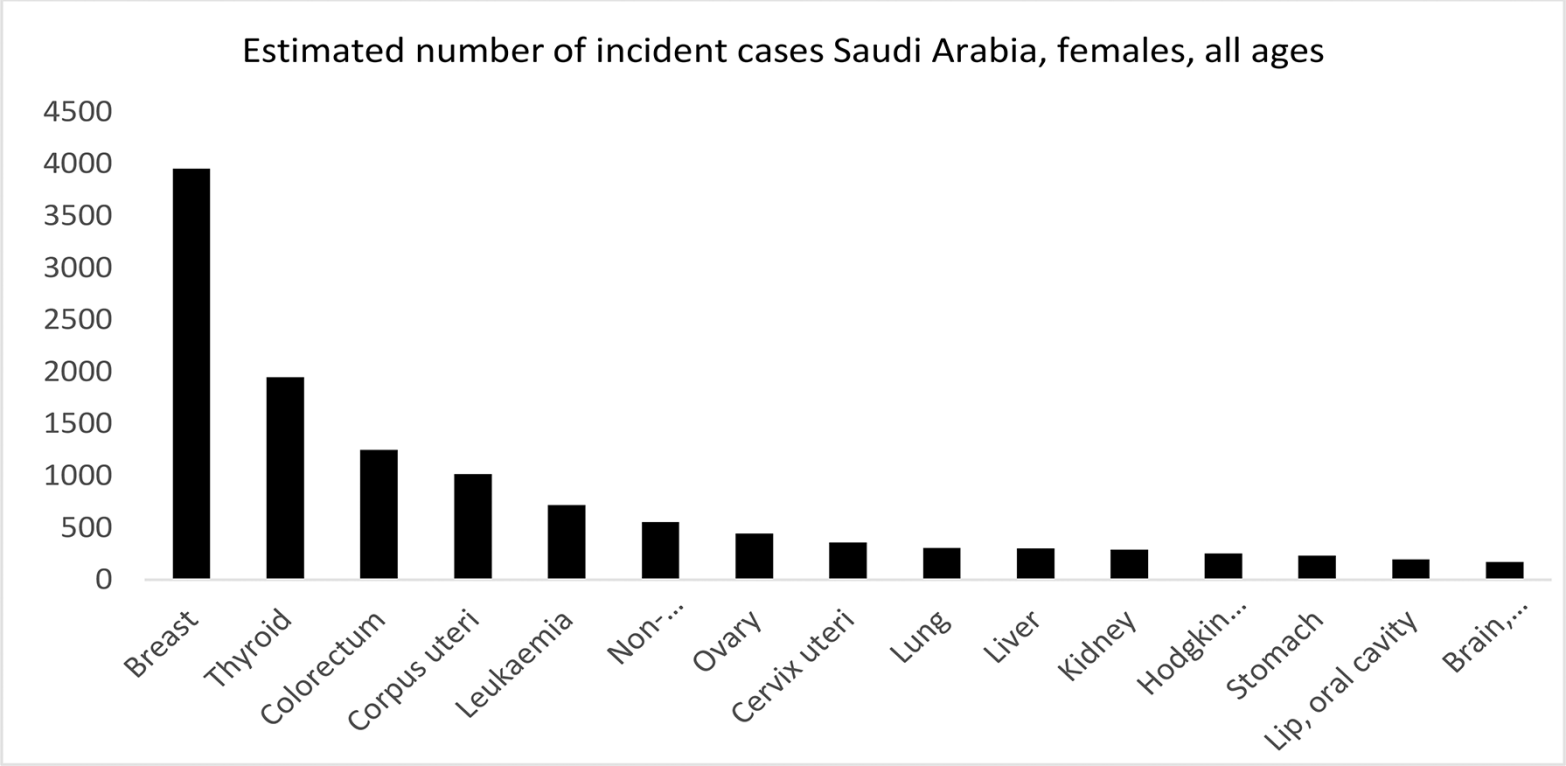
Estimated incident cases in Saudi Arabia, Females (all ages).

**Graph 6:**
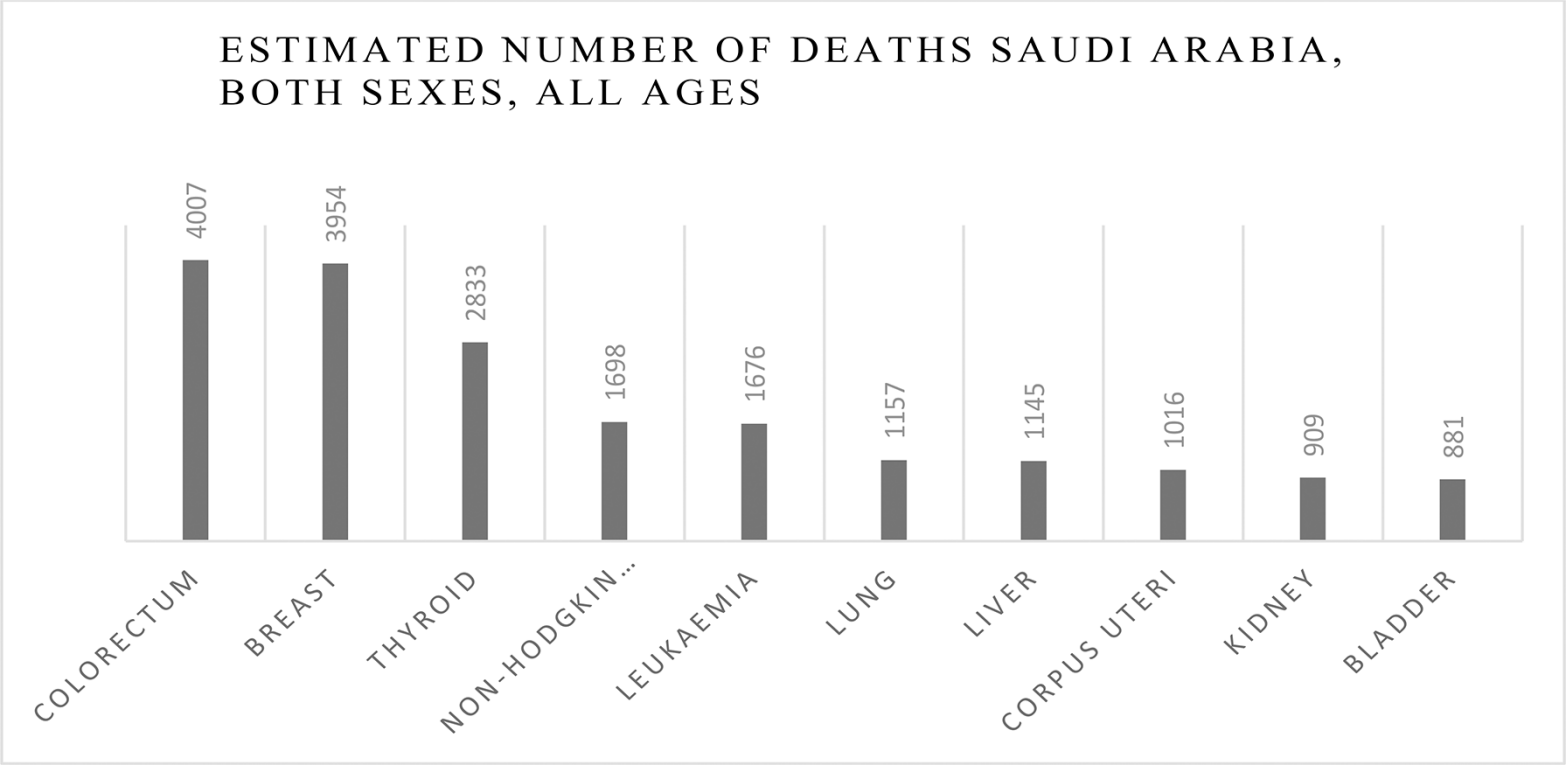
Number of deaths due to breast cancer in Saudi Arabia, all ages, both sexes.

Although detection of breast cancer in early stages has reduced morbidity and mortality rates, there is still a high rate of BC incidence among the Arab population. In 2020, there were 13,069 cancer deaths with 27,885 new cancer cases in Saudi Arabia (total population = 34,813,867) and the number of prevalent cases (5-year) was 82,640. GLOBOCAN reported that BC is the most common type of malignancy with 3,954 new cases (14.2%) during 2020 and the second type of death cause in 2020 (**Graphs 5**, **6**). In 2020, the incidence of breast cancer among females was 29% in Saudi Arabia. It is the most common cancer death in women, followed by thyroid cancer and colon cancer (**Graph 6**).

## 4 Incidence of Triple Negative Breast Cancer Around the Globe and in Saudi Arabia

Triple-negative breast cancer (TNBC), defined by the absence of estrogen receptor, progesterone receptor, and overexpression of human epidermal growth factor receptor 2 gene (HR−/HER2−), is a particularly aggressive subtype of breast cancer. Triple-negative (TN) tumors are positive for cytokeratin (CK) 5/6. They are usually high grade, with a high risk of relapse within the first few years after early diagnosis. TNBC has a prognostic value in terms of tumor size and p35 status (16, 17). For the identification of TNBC, the absence of estrogen receptor, progesterone receptor, HER2 and positivity for CK 5/6 and p53 are used as specific markers.

TNBC accounts for 15% of breast cancers diagnosed around the globe, which amounts to almost 300,000 cases each year (18). Compared with Luminal A, TNBC is more commonly diagnosed in women younger than the age of 40 (19).

**Graph 8:**
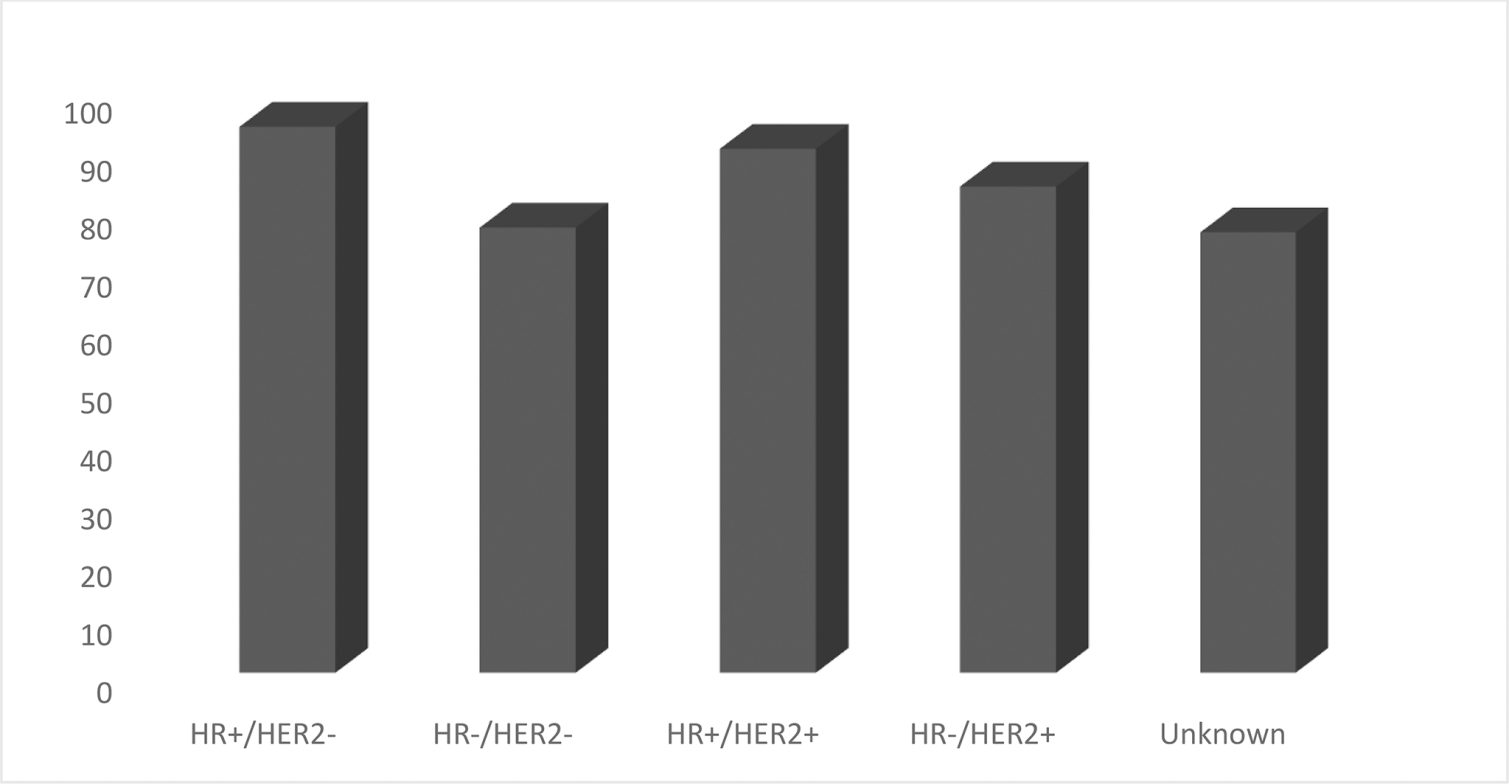
5 Year Relative Survival Percent, Female Breast Cases by Cancer Subtype (Total 5-Year Relative Survival 90.3%).

According to the Surveillance, Epidemiology, and End Results (SEER) registries for 5-year breast cancer-specific survival, TNBC is in the fourth position with 76.9% vs 94%, 90%, and 84% for Luminal A, Luminal B, and HER2+, respectively **(** **Graph 8**).

Among the Saudi population, Luminal A is the most common subtypes, followed by triple-negative tumors. The frequency of TNBC is high among African, Omani, and Tunisian communities. According to one more study by Tammimi et al. (20) TNBC is the most common subtype of BC in SA, which is a bit different compared to other studies, as most of the studies done in SA clearly indicate that Luminal-A stands in the first position.

Luminal-A was found to be the most common subtype of BC with 59% of cases in Saudi Arabia, according to a study in the western region of the kingdom. In contrast, TNBC and the Her2 subtype were the least common types of BC in the Saudi population, with 16 and 11.4%, respectively (12). According to some studies conducted in the Riyadh region in Saudi Arabia between 2010 and 2014, the triple-negative breast cancer incidence rate was found to be 14.8%. These results are in accordance with previous retrospective analysis conducted in King Abdul-Aziz Medical City (KAMC), Saudi Arabia, which states that only 12% were diagnosed with TNBC (62 patients from 514 BC cases) (17).

**Graph 9:**
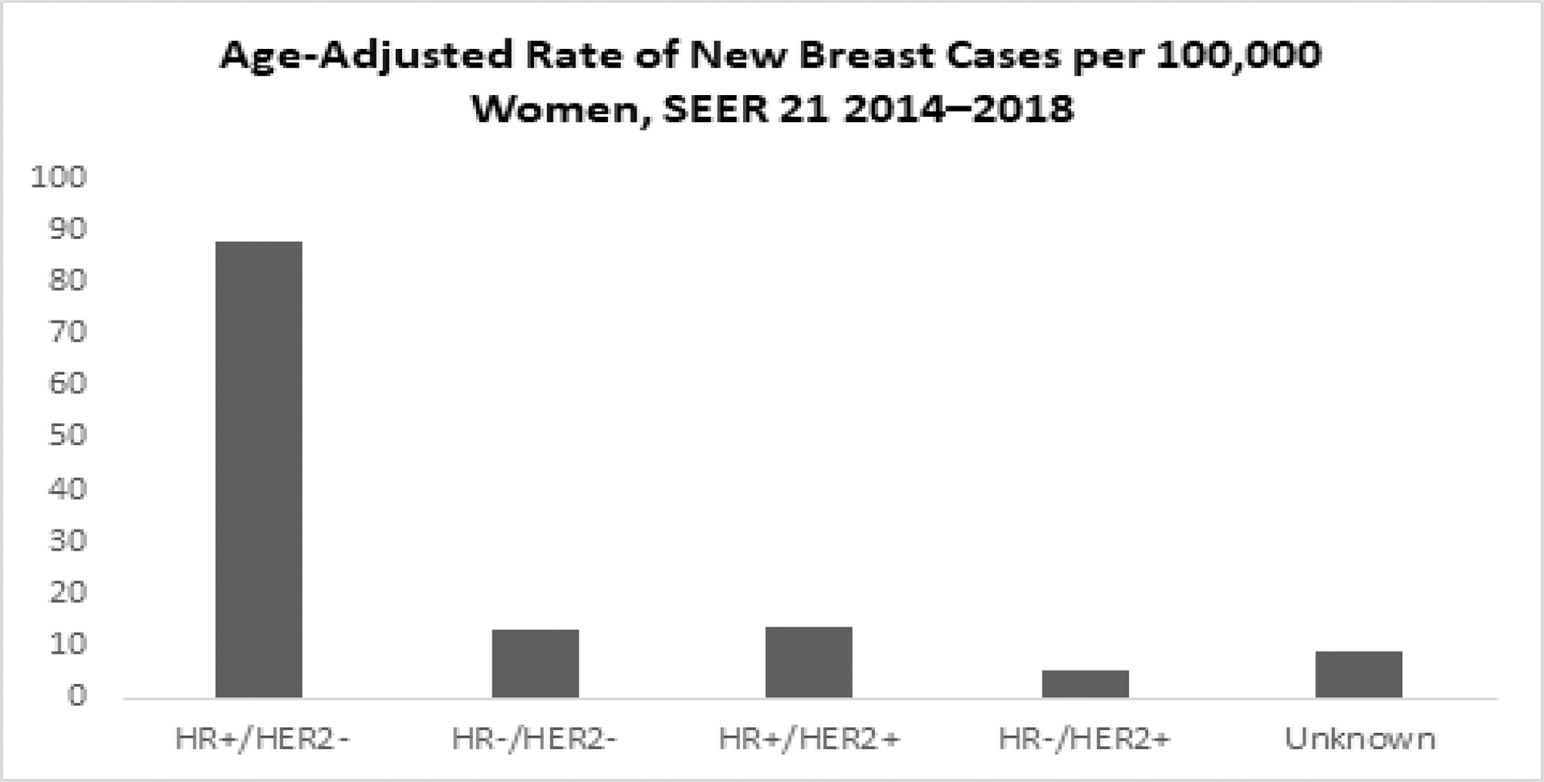
Age-Adjusted Rate of New Breast Cases per 100,000 Women (All rates are age-adjusted) (SEER 21 2014–2018).

According to SEER statistics, based on 2014–2018 cases, HR+/HER2− is the most common subtype, with an age-adjusted rate of 88.1 new cases per 100,000 women. The rate of the HR+/HER2− subtype is six times higher than the triple-negative breast cancer rate (13.1) and the HR+/HER2+ breast cancer rate (13.4), and more than 16 times higher than the HR−/HER2+ breast cancer rate (5.5) around the world (**Graph 9**).

The features of TNBC have been extensively studied in Western populations, but there are fewer studies of TNBC among non-western ethnics, especially among the Saudi population. According to a study conducted in King Abdulaziz Medical City (KAMC), Riyadh, Saudi Arabia, while comparing TNBC and non-TNBC patients, a high percentage (87.3%) of patients with TNBC expressed grade 3 tumors compared to the control. This percentage is like the others that have been reported in other places (17, 21), and these findings state that TNBC tumors are high-grade tumors. Also, TNBC is diagnosed at a later stage because of its aggressiveness and highly proliferating nature (22).

## 5 Nanocarrier Mediated Treatment of Triple Negative Breast Cancer

### 5.1 Nanomedicines and Its Applications in TNBC Treatment

Currently, triple-negative breast cancer (TNBC) does not respond to many targeted drugs due to the lack of progesterone receptor, estrogen receptor, and human epidermal growth factor receptor-2, which makes the detection and treatment of TNBC difficult and challenging. Metastatic relapse and drug resistance to chemotherapy are the major obstacles to treating triple-negative breast cancer effectively. During the last decade, investigators developed various nanomaterials that are considered a new field of science dealing with dimensions on the nanoscale. Using nanotechnology, scientists have developed various nanoparticles (NPs) for breast cancer diagnosis and therapy (Mahsa Keihan 23). Nanomedicines could help overcome the limitations of conventional therapy such as chemotherapy, radiation therapy, etc. (24). The size of the particles ranges between 10 and 100 nm and can be composed of different materials, including metals (gold, silver, and iron), lipids, polymers, silica, protein/peptides, and oligonucleotides (Jaison 25). NPs have gained popularity due to emerging biomedical applications. It can be employed in drug delivery approaches, chemical and biological sensing, gas sensing, and CO2 capture (26–33).

Various nanoparticle-formulated medications are currently being used in clinical trials to treat breast cancer (**Table 2**).

**Table 2:** Different types of nanodrugs.

Type of Nanocarriers	References
Liposomes	Alavi et al. (34) Wu et al. (35) Christensen etal. (36)
Carbon-60 fullerenes	Lin et al. (37) Wang et al. (38)
Polymer-based platforms	Wang et al. (39); Bai, X. Zhang et al. (40)
Metallic Nanoparticles: ex Gold nanoshells	Xu et al. (41) Park et al. (42)
Dendrimers	Sharma et al. (43) Lu et al. (44)
Superparamagnetic nanoparticles	Mosafer et al. (45)
Nanocrystal	A Fuhrmann et al. (46)
Silicon and silica-based nanoparticle	Meka et al. (47) Rosenbrand et al. (48)
Lipid based drug delivery	Yingchoncharoen et al. (49)
Nucleic Acid based therapeutics	Sakib Haque and Sahay (50)

As cancer is one of the most dreaded diseases, causing most deaths worldwide, more than 12,000 researchers around the world in the last decade have focused on new therapies to target cancer tissues using nanomaterials as drug delivery agents (51).

The major type of nanoparticle used to target cancer cells is coupled to chemotherapeutic drugs, while few studies have focused on the use of nanotechnology in the immunotherapy context to not only induce the cytotoxic effect on cancer cells but also treat and control breast cancer (52).

Recently, several nanomedicine studies have been conducted to specifically target specific pathways involved in cell proliferation and migration in breast cancer (**Table 3**).

**Table 3:** Clinical trials in the area of nanotechnology and triple-negative breast cancer.

Name of the study	Interventions	Phase	Trial
Carboplatin and paclitaxel albumin-stabilized nanoparticle formulation before surgery in treating patients with locally advanced or inflammatory triple negative breast cancer	Drug: carboplatin Drug: paclitaxel albumin-stabilized nanoparticle formulation Other: laboratory biomarker analysis	II	NCT01525966
Study to evaluate CORT125134 with nab-paclitaxel in patients with solid tumors	Drug: CORT125134 with nab-paclitaxel	I/II	NCT02762981
Weekly and Every 3-Week Administration of Paclitaxel Liposome Injection in Metastatic Breast Cancer	Drug: paclitaxel liposome injection	IV	NCT02142790
Veliparib in treating patients with malignant solid tumors that do not respond to previous therapy	Drug: veliparib	I	NCT00892736
Neoadjuvant pembrolizumab(Pbr)/Nab-paclitaxel followed by pbr/epirubicin/cyclophosphamide in TNBC	Pembrolizumab, Nab-paclitaxel, Epirubicin, Cyclophosphamide	II	NCT03289819
AZD2281 plus carboplatin to treat breast and ovarian cancer	Drug: AZ2281+carboplatin	I	NCT01445418
Evaluate the Efficacy and Safety of Genexol^®^-PM Compared to Genexol^®^ in Recurrent or Metastatic Breast Cancer	Drug: Genexol-PM Drug: Genexol	III	NCT00876486

**Figure 1:**
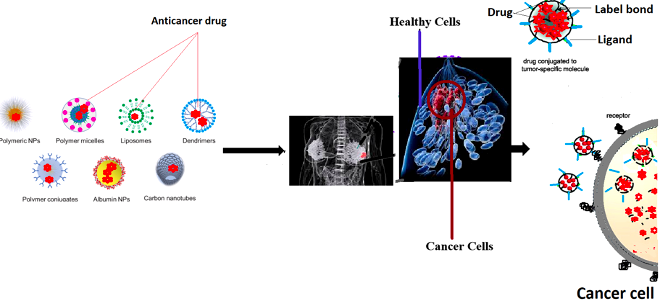
Various Nanoparticles to treat Breast Cancer.

Targeting breast cancer cells by specific nanoparticles depends on the recognition of specific molecules (ligands) on their surface and their interaction with a specific marker expressed on the surface of targeted breast cancer cells (53). These specific receptors facilitate the internalization of nanoparticles through endocytosis, and then by lysosome degradation the biomolecules are released (53) (**Figure 1**).

Various nanocarriers used for treatment of TNBC are as follows.

#### 5.1.1 Liposomes

**Figure 2:**
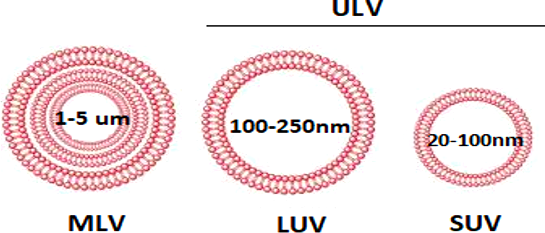
Classification of liposomes based on lamellarity.

Alec D. Bangham first described liposomes in 1961 as spherically shaped microscopic vesicles. The word liposome derives from two Greek words: lipo meaning “fat” and soma meaning “body.” The size of liposomes can vary from 100 to 400 nm vesicles. It is so named because it consists of a central aqueous core surrounded by lipid bilayers of phospholipid, which closely resemble the structure of cell membranes (**Figure 2**) and an aqueous phase. The phospholipid bilayer of the liposomes can carry lipophilic drugs, while the aqueous phase can carry hydrophilic drugs.

Liposomes are considered the most versatile nanocarriers, which makes them an attractive alternative for researchers because of their good drug distribution. There are many types of liposomes that are designed by different methods (extrusion, solvent injection, and reverse-phase evaporation), and their nomenclature depends on their method of preparation, special functions, or their structural parameters. The difference between each type of liposome is mainly related to their structure, size, surface charge, lipid composition, and vesicle dimensions. Based on the number of bilayers, liposomes can be classified into two categories: Multilamellar vesicles (MLV) with several lamellar phase lipid bilayers and unilamellar vesicles (ULV) with a single phospholipid bilayer sphere enclosing the aqueous solution. They can be further classified into two subtypes: large unilamellar vesicles (LUV) and small unilamellar vesicles (SUV) (**Figure 2**).

Based on size, liposomes are classified as

* multilamellar vesicles (MLV; >0.5 um),
* large unilamellar vesicles (LUV >100 nm)
* small unilamellar vesicles (SUV, 20–100 nm),
* Oligolamellar vesicles (OLV >0.1–1 um)
* Unilamellar vesicles (UV all range size)
*Giant unilamellar vesicles (GUV >100 um)
* Multivesicular vesicles (MV >1 um)

Based on composition, liposomes are classified as conventional liposomes (CL), PH-sensitive liposomes, cationic liposomes, long-circulating liposomes (LCL), and immuno-liposomes.

Based on the method of preparation, they are further classified as reverse phase evaporation vesicles (REV), French press vesicles (FPV), and ether injection vesicles (EIV).

#### 5.1.2 Drug delivery by Liposomes

##### 5.1.2.1 Steps of Drug Delivery by Liposome

**Figure 3:**
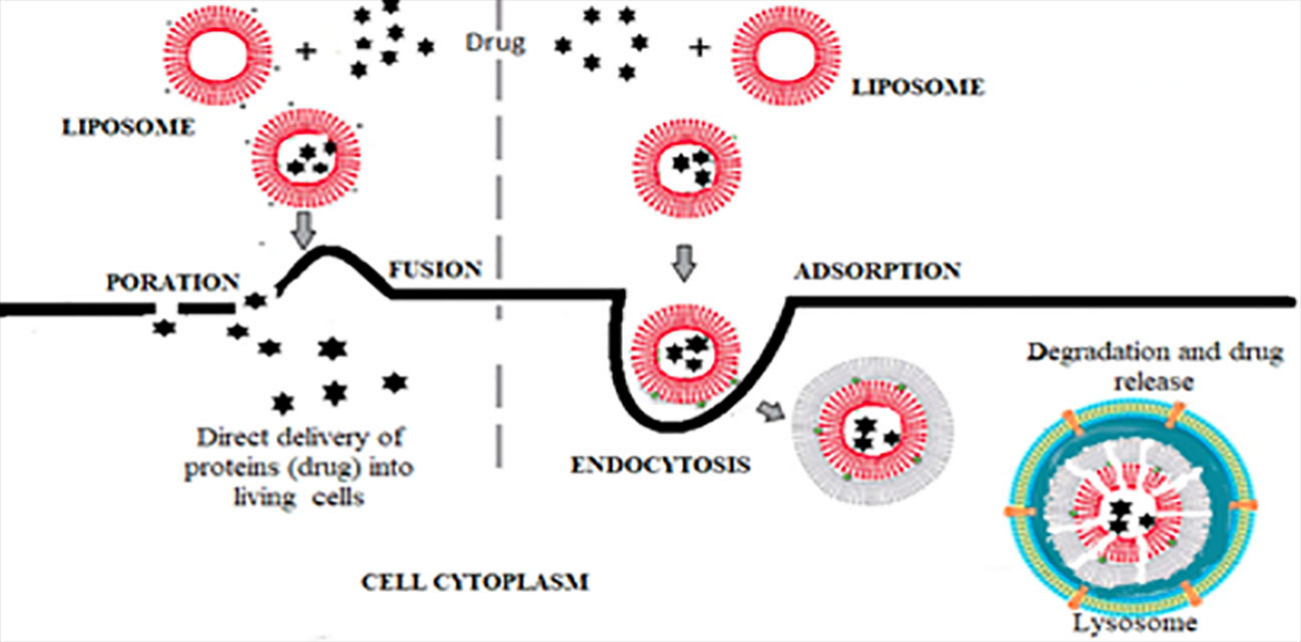
Steps of drug delivery by liposomes either by fusogenic liposome or by endosomal degradation.

**A. Adsorption:** the specific structure of liposomes as a phospholipid bilayer can mimic the lipid bilayer membrane of cells and bind to tumor cells.
**B. Endocytocis:** internalization of liposomes by the cell.
**C. Fusion:** liposomes can fuse with the cell membrane by lateral diffusion, causing the delivery of liposomal contents into the cytoplasm.
**D. Drug delivery**: The delivery of drugs into living mammalian cells can be by direct delivery of liposomal content after its fusion with the cell membrane (fusogenic liposome) or *via* endocytosis, where the liposome is degraded before reaching its destination (endosomal degradation) (54) (**Figure 3**).

#### 5.1.3 Currently Used Liposome in Triple-Negative Breast Cancer Treatment

Due to the lack of specific cellular receptors on TNBC tumor cells (ER, PR, and HER2), the aggressive nature of developing multidrug resistance (MDR) to conventional chemotherapeutics, and the metastatic potential, makes drug delivery to the tumor cells challenging (55).

The liposome is one of the most common types of nanocarrier used currently to treat TNBC because of its ability to load drugs into the aqueous core or the lipid bilayer, making it an attractive alternative in clinical trials.

##### 5.1.3.1 Clinical Trials

Liposomes approved by the FDA have been employed to deliver several chemotherapeutic drugs to treat TNBC patients, such as doxorubicin, an anthracycline type of chemotherapy that is used to treat several different types of cancer (Doxil^®^, Lipodox^®^, and Myocet^®^) (56), paclitaxel Lipusu^®^, approved in China, and daunorubicin (DaunoXome^®^), which is currently in advanced clinical trials for metastatic breast cancer (57).

Another liposomal paclitaxel (PTX) developed by MediGene known as EndoTAG-1^®^ is positively charged, which increases the permeability of cancer cells and enhances the uptake of anticancer drugs by tumor cells (58).

Awada and coworkers evaluated the antitumor efficacy of EndoTAG with Taxol^®^ in 140 patients in phase II clinical trial (NCT00448305) (59). They stated that EndoTAG-Taxol significally decreases the tumor proliferation and cell toxicity in healthy cells.

PTEN (phosphatase and tension homolog) is a tumor suppressor frequently decreased in TNBC cells. The loss of PTEN activates mTOR (mammalian target of rapamycin (mTOR), which is a key target for anticancer therapy in TNBC (60 *).*

The inhibition of mTOR by a specific molecule such as Rapamycin (RAPA) has been clinically proven. RAPA shows anti-tumor activity against TNBC, but it can induce drug resistance when used alone (61)*. *Recently, using liposomal DOX in a phase I trial in TNBC patients, Weinbeg et al. stated that liposome DOX-RAPA inhibited the upregulation of hypoxia-inducible factor 1-alpha (HIF-1α), which is a transcription factor highly expressed in tumor proliferation, migration, and drug resistance.

#### 5.1.4.Liposomes Used in Saudi Arabia to Treat TNBC

In addition, Lamyaa et al. published a recent study evaluating the efficacy of the new miR-1296 liposome in TNBC [Lamyaa (62)]. miR-1296 is a novel cancer-related miRNA found tp be dysregulated in various cancers, gastric cancer, prostate cancers, cervical cancer, and TNBC (63, 64). The downregulation of miR-1296 can enhance tumor cell proliferation, but its re-expression blocks cell invasion and metastasis. The efficacy of naked miRNAs evaluated in several studies is still limited due to their short half-life *in vivo*, biological instability, poor penetration, and rapid clearance by cells (65, 66). The aim of Lamyaa and colleagues was to develop a cationic nanoliposome delivery system for miR-1296 and miR-1269 and to evaluate its cellular uptake and its effect on TNBC cell culture. It was shown that upregulation of miR-1269 inhibits TNBC cell proliferation and promotes cell apoptosis. They demonstrated that the miR-1269 liposome is one of many other miRNAs that can be used in clinical applications of TNBC therapy.

Several deregulated tumor-suppressive miRNAs were involved in cells in TNBC. For instance, *MiR-203* is one of the well-described microRNAs that is downregulated in TNBC cells. In addition, mir-200c, miR-205, and mi-206 were found to be downregulated in TNBC and their upregulation was correlated with the inhibition of cell migration and tumor growth (38, 67, 68).

The interesting fundamental role of miRNA as a tumor-suppressor made it a good approach to be used along with nanotechnology making a new technology for treating TNBC.

The use of nanocarriers in clinical trials for TNBC is still limited in Saudi Arabia, but many clinicians are still working to evaluate the efficacy of liposomes as a target approach to treat several types of cancers, such as Masood Khan et al., who stated that glycosphingosomes significantly reduce the tumor profile and induce apoptosis.

### 5.2 Metallic Nanoparticles

Metallic nanoparticles (MNPs) are extremely useful in cancer treatment. Nanoparticles can be classified into 2 categories: organic and inorganic NPs. Metallic NPs are listed as inorganic NPs; however, liposomes, micelles, dendrimers, and polymeric NPs are considered organic NPs (69) (**Table 4**).

**Table 4:** Showing different types of metallic nanoparticles (MNPs) and their composition.

Type of Metallic NP	composition	Elements
Metal-ion NPs	originated from the metal ion itself	Ag, Au, Pt, Ni, Cu
Metal oxide NPs	consist of metal ions in the oxide forms	CuO, ZnO, TiO2, CeO2, SiO2
Metal sulfide NPs	NP in sulfide form	CuS, Ag2S,FeS
Bimetallic NPs.	NP developed from two metals forms a bimetallic NP	FePt, FeCo, FeNi, CuNi

Metallic nanoparticles can be classified into 4 major subtypes, including metal-ion NPs, metal oxide NPs, metal sulfide, and bimetallic NPs. The first category of nanomaterial called metal nanoparticles includes different types of metals such as gold (Au), copper (Cu), silver (Ag), titanium (Ti), platinum (Pt), magnesium (Mg), and zinc (Zn) nanoparticles. The metal oxide nanoparticles are a specific group including titanium dioxide, silver oxide, and zinc oxide, which are widely used in therapeutic trials (70). Moreover, metal sulfide nanomaterials (e.g., AgS, CuS, and FeS) are used in various medical applications because of their excellent antimicrobial activities. Bimetallic NPs, as the name suggests, are composed of two different metal elements, such as CuS, Ag2S, FeS, etc.

The most extensively explored metallic nanoparticles in biological applications such as cancer therapy, diagnosis, and drug delivery are gold (Au NPs) (71) and silver NPs (72).

#### *5.2.1* Gold Nanoparticle and TNBC

Gold nanoparticles (AuNPs) have low toxicity and have the ability to attach to anti-cancer drugs such as paclitaxel. Recently used *in vivo*, the combination of paclitaxel and AuNPs enhanced the efficiency of drug delivery to target cells and reduced BC cell proliferation.

This main idea was supported by Kumar et al. They used paclitaxel and curcumin (Cur) along with gold NP to evaluate the anti-metastatic activity of this chemotherapeutic drug in various *in vitro* and *in vivo* models of TNBC. They suggested that Au NPs downregulate the expression of VEGF, CYCLIN-D1, and STAT-3 genes and upregulate the apoptotic caspase-9 gene.

The first group of mice that received paclitaxel and curcumin with gold nanoparticles showed a significant reduction in the size of TNBC tumors compared with the second group treated with Cur–paclitaxel alone. Also, Kong et al. stated that gold nanoparticles have little or no toxicity to healthy cells, but they can significantly enhance cancer killing and reduce cell migration and invasion by reducing the rate of energy (73).

Additionally, Andey et al. (74) suggested that using the combination AuNPs–cisplatin, a chemotherapeutic drug, significantly reduces the TNBC proliferation and metastasis.

#### 5.2.2 Silver Nanoparticles and TNBC

Silver nanoparticles (AgNPs) have been extensively used because of their antimicrobial properties and their cytotoxic effects against cancer cells, including breast cancer, ovary cancer, brain cancer, liver cancer, colon cancer, and blood cancer (75–78).

A recent research published by Swanner and colleagues stated that TNBC and nonmalignant breast cells both uptake AgNPs and AgNPs are extremely lethal for TNBC cells because of their rapid degradation in cancer cells. However they were not cytotoxic to healthy cells (79). After the internalization of AgNPs, the intercellular rate of stress increased due to the high synthesis of cellular antioxidants, which stresses the endoplasmic reticulum in TNBC cells but does not harm normal cells in the same way.

*In vivo*, the same team showed for the first time that systemically administered AgNPs but can significantly reduce the growth of solid TNBC tumors in mice. However, clinical trials in TNBC patients using AgNPs are still limited.

### 5.3 Nucleic Acid-Based Therapeutics

Nucleic acids (NA) are the most important information-carrying molecules in all cells and viruses. The main function of NA is to control the expression of genetic information and protein synthesis. Deoxyribonucleic acid (DNA) and ribonucleic acid (RNA) are two natural types of nucleic acids. Therapeutic nucleic acids (TNAs) are nucleic acids themselves or modified RNA or DNA, which have recently proven to be a valuable tool to modulate gene expression (80).

Although various types of TNAs exist, they have successfully proved their ability to target diseases at the genetic level by down or upregulating numerous proteins, which are related to cell proliferation or apoptosis, respectively. The development of nucleic acid (NA)-based therapy, widely explored in the last decade to treat diseases, has produced two independent new strategies, such as DNA and RNA nanotechnology (81).

Targeting the intrinsic cancer pathways altered during tumorigenesis makes RNA-based therapeutics a good tool in the clinic (81). Currently, two major types of RNA-based therapy are used in clinical trials; interference (RNAi), used usually in gene silencing to control cell growth and death; and RNA nanotherapy, widely used in drug delivery to target cells.

RNA interference can be used as a therapeutic tool in two ways: either small interfering RNA (siRNA) or microRNA (miRNA) (82). MicroRNAs can play crucial roles in gene regulation and expression in TNBC. Recently, (82) showed that RNA nanotechnology has clinical applications to deliver miRNA-based therapeutics for TNBC. They used miR-21, an oncogene involved in tumor progression and metastasis in several cancers, including TNBC. They used RNA nanotechnology to deliver mi-ARN and anti-miRNA to cancel cells without causing any damage to healthy cells.

### 5.4 Dendrimers

Dendrimers are highly branched and symmetrical nanosized agents with 2 to 10 nm in diameter. They possess the unique property of being monodisperse and homogeneous, which makes them ideal nanocarrier-based systems for cancer chemotherapy. They work in cancer therapy *via* ligand and receptor-mediated endocytosis. In MCF-7 cells, PAMAM dendrimers loaded with functional siRNA are used to target the MDR1 gene, which is responsible for drug resistance development. According to one study, a nanocomplex known as dendriplexes was generated using a phospholipid (PL) modified PAMAM–siMDR1 complex. This complex showed considerable gene silencing, increased siMDR1 uptake and decreased P-gp expression, resulting in increased DOX cellular accumulation (83). In another study, the CXCR4 gene was targeted and inhibited on the surface of BT-549 triple-negative breast cancer cells by developing PAMAM dendrimers encapsulating DOX modified with LFC131 peptide (84). Various scientists have used PPI dendrimers in drug delivery for and against MCF-7 cells for treating breast cancer. Kaur et al. created folate-conjugated polypropylene imine dendrimers to effectively deliver methotrexate (MTX) to MCF-7 cells.

### 5.5 CRISPR Nanoparticles

Clustered regularly interspaced short palindromic repeats (CRISPR)/associated (Cas9) technology is being widely used along with genome engineering to overcome drug resistance in breast cancer chemotherapy. The CRISPR technology reverses genetic alteration, the major contributing factor in drug-resistance in cancer chemotherapy, by recognizing the possible target areas causing this resistance to drugs. To repair the genetic information in TNBCs, various nanoparticulate systems encapsulated in nanogels have been investigated using CRISPR technology (85). CRISPR/Cas9 is a gene-editing technology that can correct genomic faults. It is a rapid method for activating or deactivating certain genes in cells. CRISPR/Cas9 technology has proven to be an effective technique for treating various genetic diseases, including human breast cancer. Liposome-based hydrogel nanoparticles (LHNPs) have been drafted to convey Cas9 protein and nucleic acids, and they might be programmed to stop genes from being expressed in cancers (63). Thus, CRISPR cab be employed for both experimental and clinical gene therapy in cancer.

### 5.6 Lipid-Based Nanomedicines

Lipid-based colloids are biodegradable and biocompatible with biomolecules such as phospholipids, cholesterol, and triglycerides, and thus can be used to target different cancerous cells at the same time when constructed as nanostructured lipid carriers. These lipid-based carriers can be made from a variety of materials and have a wide range of therapeutic and diagnostic applications in cancer research. According to Sun et al., nanostructured lipid carriers (NLCs) loaded with Quercetin reduce the solubility problems of Quercetin and the efficacy of entrapment was found to be 95%, while its extended-release resulted in cell killing in MCF-7 and MDA-MB-231 cells (86). Also, DOX and PTX when encapsulated with NLCs target MCF-7, SK-OV3 cell lines, and their other variants. Tamoxifen citrate and camptothecin with solid lipid nanoparticles (SLNs) showed an advantage against MCF-7 and MCF-10A cells (87). Solid lipid nanoparticles (SLNs) have various advantages like protection of drug from degradation, long-term stability, reduce systemic toxicity, are feasible to load lipophilic and hydrophilic drugs, and are easy to scale up, with some disadvantages as well, which include causing unpredictable agglomeration, having a high incidence of polymorphic transition, and drug expulsion (88). They also carry desirable particle sizes. Lipid polymer hybrid nanoparticles are also considered to be reliable lipid-based nanocarriers. They have provision for surface modification and dose entrapment of hydrophilic and lipophilic drugs with high serum stability (89). According to a study by (90) in 2012, the drug mitoxantrone, which is water-soluble, was encapsulated into lipid polymer nanoparticles (LPNs). Mitoxantrone LPN increased cytotoxic action than the drug alone in the MCF-7 and MCF-7-MX. Many other nanoparticles like carbon nanoparticles, exosomes, natural agent based nano-carriers, inorganic nanoparticles, and polymeric micelles are also being used as promising treatments for breast cancer and its subtypes like TNBC.

## 6 Conclusion

TNBC is less studied among non-western ethnic groups, especially among the Saudi population. Also, TNBC is diagnosed at a later stage because of its aggressiveness and highly proliferating nature, and while comparing TNBC and non-TNBC patients, a high percentage of patients with TNBC expressed grade 3 tumors in Saudi Arabia. Nanomedicine aids in the diagnosis and treatment of both hormone-negative and hormone-positive breast cancer and is said to be one of the most promising options among the various treatment methods available for treating TNBC. A wide variety of nanoparticle-centric drug formulations have been developed and are already playing a vital role in treating the disease. Various nanocarriers like liposomes, metallic nanoparticles, dendrimers, nucleic acid-based nanomaterials, CRISPR nanoparticles, lipid-based nanomedications, and others are being extensively used in the treatment of breast cancers like TNBC. In the future, a greater understanding of nanotechnological improvements will undoubtedly aid in the development of more suitable nanomedicines for treating breast cancers.

## Author Contributions

IN wrote introduction and incidence of TNBC. SBE compiled the data of trials in treatment of TNBC and nanomedicines used in its treatment while SBI and SC helped in manuscript preparation and analysis of all the data. All authors listed have made a substantial, direct, and intellectual contribution to the work and approved it for publication.

## Funding

The authors extend their acknowledgment to the Deanship of Scientific Research at King Faisal University, Al Hofuf, Saudi Arabia, under project number AN000480 for the financial support.

## Conflict of Interest

The authors declare that the research was conducted in the absence of any commercial or financial relationships that could be construed as a potential conflict of interest.

## Publisher’s Note

All claims expressed in this article are solely those of the authors and do not necessarily represent those of their affiliated organizations, or those of the publisher, the editors and the reviewers. Any product that may be evaluated in this article, or claim that may be made by its manufacturer, is not guaranteed or endorsed by the publisher.

## References

[1] Hassanpour SH Dehghani M. Review of Cancer from Perspective of Molecular. J Cancer Res Pract (2017) 4(4):127–9. doi: 10.1016/j.jcrpr.2017.07.001

[2] Hou K Ning Z Chen H Wu Y. Nanomaterial Technology and Triple Negative Breast Cancer. Front Oncol (2022) 11:828810. doi: 10.3389/fonc.2021.828810 35096628 PMC8790081

[3] El-Sahli S Hua K Sulaiman A Chambers J Li L Farah E. A Triple-Drug Nanotherapy to Target Breast Cancer Cells, Cancer Stem Sells, and Tumor Vasculature. Cell Death Dis (2021) 12(1):8. doi: 10.1038/s41419-020-03308-w 33414428 PMC7791049

[4] Alnegheimish NA Alshatwi RA Alhefdhi RM Arafah MM AlRikabi AC Husain S. Molecular Subtypes of Breast Carcinoma in Saudi Arabia. A Retrospective Study. Saudi Med J (2016) 37(5):506–12. doi: 10.15537/smj.2016.5.15000 27146612 PMC4880649

[5] Goldhirsch A Wood WC Coates AS Gelber RD Thürlimann B Senn H-J. Strategies for Subtypes–Dealing With the Diversity of Breast Cancer: Highlights of the St. Gallen International Expert Consensus on the Primary Therapy of Early Breast Cancer 2011. Ann Oncol (2011) 22:1736–47. doi: 10.1093/annonc/mdr304 PMC3144634 21709140

[6] Prat A Perou CM. Deconstructing the Molecular Portraits of Breast Cancer. Mol Oncol (2011) 5:5–23. doi: 10.1016/j.molonc.2010.11.003 21147047 PMC5528267

[7] Abulkhair O Saadeddin A Makram O Gasmelseed A Pasya T Sehata H. Vitamin D Levels and Breast Cancer Characteristics: Findings in Patients From Saudi Arabia. J Steroid Biochem Mol Biol (2015) 164:106–9. doi: 10.1016/j.jsbmb.2015.11.003 26554935

[8] Rahman S Zayeh H. Breast Cancer in the GCC Countries: A Focus on BRCA1/2 and non-BRCA1/2 Genes. Genes (2018) 688:73–76. doi: 10.1016/j.gene.2018.05.045 29777908

[9] Zayed H. The Arab Genome, Health and Wealth. Gene (2016) 592:239–43. doi: 10.1016/j.gene.2016.07.007 27393651

[10] Bazarbashi S Al-Eid H Minguet J. Cancer Incidence in Saudi Arabia: 2012 Data From the Saudi Cancer Registry. Asian Pacific J Cancer Prev (2017) 9:2437–44. doi: 10.22034/APJCP.2017.18.9.2437 PMC5720648 28952273

[11] Saudi Cancer Registry, Cancer Incidence Report (2019), Saudi Arabia.

[12] Al-Thoubaity FK. Molecular Classification of Breast Cancer: A Retrospective Cohort Study. Ann Med Surg (Lond) (2019) 49:44–8. doi: 10.1016/j.amsu.2019.11.021 31890196 PMC6926136

[13] Mehdi I Monem AA Al Bahrani B Ramadhan FA. Breast Cancer Molecular Subtypes in Oman: Correlation With Age, Histology, and Stage Distribution - Analysis of 542 Cases. Gulf J Oncolog (2014) 1(15):38–48. 24610287

[14] Hamadeh RR Abulfatih NM Fekri MA Al-Mehza HE. Epidemiology of Breast Cancer among Bahraini Women Data from the Bahrain Cancer Registry : Sultan Qaboos. Univ Med J (2014) 14(2):e176–82. doi: 10.1001/jamaoncol.2020.2965 PMC3997533 24790739

[15] National Cancer Institute. National Institutes of health, USA, The Breast Cancer Risk Assessment Tool. (2020).

[16] Chae BJ Bae JS Lee AW Park WC Seo YJ Song BJ. P53 as a Specific Prognostic Factor in Triple-Negative Breast Cancer. Jpn J Clin Oncol (2009) 39:217–24. doi: 10.1016/j.intimp.2020.106417 19304743

[17] Abulkhair O Moghraby JS Badri M Alkushi A. Clinicopathologic features and prognosis of triple-negative breast cancer in patients 40 years of age and younger in Saudi Arabia. Hematol Oncol Stem Cell Ther (2012) 5(2):101–6. doi: 10.5144/1658-3876.2012.101 22828374

[18] Yin L Duan JJ Bian XW. And Yu, S. C. Triple-Negative Breast Cancer Molecular Subtyping and Treatment Progress. Breast Cancer Res (2020) 22:61–13. doi: 10.1186/1471-2407-10-223 PMC7285581 32517735

[19] Singh S Numan A Agrawal N Tambuwala MM Singh V Kesharwani P. Role of Immune Checkpoint Inhibitors in the Revolutionization of Advanced Melanoma Care. Int Immunopharmacol (2020) 83:106417. doi: 10.1016/j.intimp.2020.106417 32200155

[20] Tamimi DMA Shawarby MA Ahmed A Hassan AK AlOdaini AA. Protein Expression Profile and Prevalence Pattern of the Molecular Classes of Breast Cancer - a Saudi Population Based Study. BMC Cancer (2010) 10:223. doi: 10.1186/1471-2407-10-223 20492711 PMC2880995

[21] Dent R Trudeau M Pritchard KI Hanna WM Kahn HK Sawka CA. Clin Cancer Res. (2007) 13(15 Pt 1):4429–34. doi: 10.3390/pharmaceutics13020287 17671126

[22] Khan W Ashfaq UA Aslam S Saif S Aslam T Tusleem K. Anticancer Screening of Medicinal Plant Phytochemicals Against Cyclin-Dependent Kinase-2 (CDK2): An In-Silico Approach. Advance in Life sciences (2017) 4:113–9. doi: 10.3762/bjnano.9.98

[23] Shokooh MK Emami F Jeong J-H Yook S. Bio-Inspired and Smart Nanoparticles for Triple Negative Breast Cancer Microenvironment. Pharmaceutics (2021) 13:287. doi: 10.1021/ar2000259 33671698 PMC7926463

[24] Khan I Saeed K Khan I. Nanoparticles: Properties, Applications and Toxicities. Arabian J Chem (2019) 12(7):908–31. doi: 10.1016/j.arabjc.2017.05.011

[25] Jeevanandam J Barhoum A Chan YS Dufresne A Danquah MK. Review on Nanoparticles and Nanostructured Materials: History, Sources, Toxicity and Regulations. Beilstein J Nanotechnol (2018) 9:1050–74. doi: 10.3762/bjnano.9.98 PMC5905289 29719757

[26] Lee JE Lee N Kim T Kim J Hyeon T. Multifunctional Mesoporous Silica Nanocomposite Nanoparticles for Theranostic Applications. Acc Chem Res (2011) 44:893–902. doi: 10.1021/ar2000259 21848274

[27] Barrak Saied T Chevallier P Laroche G M’nif A Hamzaoui.Synthesis AH. Characterization, and Functionalization of ZnO Nanoparticles by N-(Trimethoxysilylpropyl) Ethylenediamine Triacetic Acid (TMSEDTA): Investigation of the Interactions Between Phloroglucinol and ZnO. Arab J Chem (2016) 12:4340–7. doi: 10.1016

[28] Mansha M Qurashi A Ullah N Bakare FO Khan I Yamani ZH. Synthesis of In2O3/graphene Heterostructure and Their Hydrogen Gas Sensing Properties Ceram. Int (2016) 42:11490–5. doi: 10.1016/j.ultsonch.2016.06.025

[29] Rawal Kaur A. Synthesis of Mesoporous Polypyrrole Nanowires/Nanoparticles for Ammonia Gas Sensing Application. Sens. Actuators A Phys (2013) 203:92–102. doi: 10.1016/j.arabjc.2013.04.031

[30] Ullah Khan I Yamani ZH Qurashi A. Sonochemical-Driven Ultrafast Facile Synthesis of SnO2 Nanoparticles: Growth Mechanism Structural Electrical and Hydrogen Gas Sensing Properties Ultrason. Sonochem (2017) 34:484–90. doi: 10.1039/C5CP03576G 27773272

[31] Ganesh Hemalatha P Peng MM Jang HT. One Pot Synthesized Li, Zr Doped Porous Silica Nanoparticle for Low Temperature CO2 Adsorption. Arab J Chem (2017) 10:S1501–5. doi: 10.1016/j.arabjc.2013.04.031

[32] Ramacharyulu PVRK Muhammad R PraveenKumar J Prasad GK Mohanty.Iron phthalocyanine modified mesoporous titania nanoparticles for photocatalytic P. Activity and CO2 Capture Applications .Phys. Chem Chem Phys (2015) 17:26456–62. doi: 10.1039/c5cp03576g 26393761

[33] Shaalan M Saleh M El-Mahdy M El-Matbouli M. Recent Progress in Applications of Nanoparticles in Fish Medicine: A Review Nanomed. Nanotechnol. Biol Med (2016) 12:701–10. doi: 10.2147/IJN.S187906 26656532

[34] Alavi Karimi N Safaei M. Application of Various Types of Liposomes in Drug Delivery Systems. Advanced Pharmaceutical Bulletin (2017) 7:3–9. doi: 10.15171/apb.2017.002 28507932 PMC5426731

[35] Wu Zhang Y Yang D Zhang J Ma J Cheng D. Novel SN38 Derivative-Based Liposome as Anticancer Prodrug: An *In Vitro* and *In Vivo* Study. Int J Nanomedicine (2019) 14:75–85. doi: 10.2147/IJN.S187906 30587986 PMC6304248

[36] Christensen Henriksen JR Jørgensen JT Amitay Y Shmeeda S Gabizon AG. Folate Receptor Targeting of Radiolabeled Liposomes Reduces Intratumoral Liposome Accumulation in Human KB Carcinoma Xenografts,” International Journal of Nanomedicine, Vol. 13 (2018) pp:7647–56. doi: 10.2147/IJN.S182579 PMC6251465 30538449

[37] Lin C-M Lu T-Y. C60 Fullerene Derivatized Nanoparticles and Their Application to Therapeutics. Recent Patents Nanotechnology (2012) 6:105–13. doi: 10.2147/IJN.S178650 22455733

[38] Wang Jin C Liang H Tang Y Zhang H Yang Y. Effects of Fullerene C(6)(0) Nanoparticles on A549 Cells. Environmental Toxicology and Pharmacology (2014) 37:656–61. doi: 10.1016/j.etap.2014.01.015 24577232

[39] Wang J Seo MJ Deci MB Weil BR Canty JM Nguyen J. Effect of CCR2 Inhibitor-Loaded Lipid Micelles on Inflammatory Cell Migration and Cardiac Function After Myocardial Infarction. Int J Nanomedicine (2018) 13:6441–51. doi: 10.2147/IJN.S178650 PMC6198873 30410330

[40] Bai S Zhang X Ma X Chen J Chen Q Shi X. Acid-Active Supramolecular Anticancer Nanoparticles Based on Cyclodextrin Polyrotaxanes Damaging Both Mitochondria and Nuclei of Tumor Cells. Biomaterials Sci (2018) 6:3126–38. doi: 10.1684/bdc.2014.1940 30362476

[41] Xu X Bayazitoglu Y Meade A Jr. Evaluation of Theranostic Perspective of Gold-Silica Nanoshell for Cancer Nano-Medicine: A Numerical Parametric Study. Lasers Med Sci (2018) 34(2):377–88. doi: 10.7150/thno.29039 30215184

[42] Park SE Lee J Lee T. Comparative Hyperthermia Effects of Silica-Gold Nanoshells With Different Surface Coverage of Gold Clusters on Epithelial Tumor Cells. Int J Nanomedicine (2015) 10:261–71. doi: 10.2147/IJN.S88309 PMC4583537 26425093

[43] Sharma A Liaw K Sharma R Zhang Z Kannan S Kannan RM. Targeting Mitochondrial Dysfunction and Oxidative Stress in Activated Microglia Using Dendrimer-Based Therapeutics. Theranostics (2018) 8:5529–47. doi: 10.1016/j.msec.2016.11.053 PMC6276292 30555562

[44] Lu S Li X Zhang J Peng C Shen M Shi X. Dendrimer-Stabilized Gold Nanoflowers Embedded With Ultrasmall Iron Oxide Nanoparticles for Multimode Imaging–Guided Combination Therapy of Tumors. Advanced Sci (2018) 5:1801612. doi: 10.1021/mp5001247 PMC6299682 30581720

[45] Mosafer Teymouri M Abnous K Tafaghodi M Ramezani M. “Study and Evaluation of Nucleolin-Targeted Delivery of Magnetic PLGA-PEG Nanospheres Loaded With Doxorubicin to C6 Glioma Cells Compared With Low Nucleolin-Expressing L929 Cells,”. Materials Sci Eng C: Materials Biol Appl (2017) 72:123–33. doi: 10.3390/pharmaceutics10040283 28024568

[46] A Fuhrmann Gauthier MA Leroux J-C. Targeting of Injectable Drug Nanocrystals. Mol Pharmaceutics. (2014) 11:1762–71. doi: 10.2147/IJN.S182428 24766270

[47] Meka Jenkins L Dàvalos-Salas M Pujara N Wong KY Kumeria T. Enhanced Solubility, Permeability and Anticancer Activity of Vorinostat Using Tailored Mesoporous Silica Nanoparticles. Pharmaceutics (2018) 10:283. doi: 10.1124/pr.115.012070 PMC6321298 30562958

[48] Rosenbrand R Barata D Sutthavas P Mohren R Cillero-Pastor B Habibovic P. Lipid Surface Modifications Increase Mesoporous Silica Nanoparticle Labeling Properties in Mesenchymal Stem Cells. Int J Nanomedicine (2018) 13:7711–25. doi: 10.2147/IJN.S182428 PMC6251437 30538454

[49] Yingchoncharoen P Kalinowski DS Richardson DR. Lipid-Based Drug Delivery Systems in Cancer Therapy: What Is Available and What Is Yet to Come. Pharmacol Rev (2016) 68(3):701–87. doi: 10.3892/or.2017.5718 PMC4931871 27363439

[50] Sakib Haque KC Sahay G. Conroy Sun,RNA-Based Therapeutics: Current Developments in Targeted Molecular Therapy of Triple-Negative Breast Cancer. Pharmaceutics (2021) 13(10):1694. doi: 10.3390/pharmaceutics13101694 34683988 PMC8537780

[51] Li Z Tan S Li S Shen Q Wang K. Cancer Drug Delivery in the Nano Era: An Overview and Perspectives (Review). Oncol Rep (2017) 38:611–24. doi: 10.2147/IJN.S140325 PMC5562049 28627697

[52] Moses S Edwards V Brantley E. Cytotoxicity in MCF-7 and MDA-MB-231 Breast Cancer Cells, Without Harming MCF-10A Healthy Cells. J Nanomedicine. Nanotechnology (2016) 7:2. doi: 10.1021/acs.langmuir.6b04304

[53] Singh SK Singh S Lillard JW Jr. Singh R. Drug Delivery Approaches for Breast Cancer. Int J Nanomedicine (2017) 12:6205–18. doi: 10.1016/j.jconrel.2012.03.020 PMC5576701 28883730

[54] Kube S Hersch N Naumovska E Gensch T Hendriks J Franzen A. Fusogenic Liposomes as Nanocarriers for the Delivery of Intracellular Proteins. Langmuir (2017) 33:1051–9. doi: 10.1021/acs.langmuir.6b04304 28059515

[55] Thakur V Kutty RV. Recent Advances in Nanotheranostics for Triple Negative Breast Cancer Treatment. J Exp Clin Canc. Res (2019) 38(1):430. doi: 10.1056/NEJMoa1814017 PMC6819447 31661003

[56] Barenholz Y. Doxil® — The First FDA-Approved Nano-Drug: Lessons Learned. J Control Release (2012) 160:117–34. doi: 10.1093/annonc/mdu025 22484195

[57] Thorat ND Bauer J. Nanomedicine: Next Generation Modality of Breast Cancer Therapeutics. In: Thorat ND Bauer J, editors. Nanomedicines for Breast Cancer Theranostics. Elsevier (2020). p. 3–16.

[58] Strieth C Dunau C Michaelis U Jäger L Gellrich D Wollenberg B. Phase I/II Clinical Study on Safety and Antivascular Effects of Paclitaxel Encapsulated in Cationic Liposomes for Targeted Therapy in Advanced Head and Neck Cancer Head Neck. Head Neck (2014) 36(7):976–84. doi: 10.1016/j.bcp.2011.11.022 23733258

[59] Awada I Bonneterre J Nowara E Ferrero J Bakshi A Wilke C. A Randomized Controlled Phase II Trial of a Novel Composition of Paclitaxel Embedded Into Neutral and Cationic Lipids Targeting Tumor Endothelial Cells in Advanced Triple-Negative Breast Cancer (TNBC). Ann Oncol (2014) 25(4):824–31. doi: 10.1016/j.jsps.2021.04.007 24667715

[60] Walsh S. , 178–82.

[61] Loehberg CR. Akt and P53 are Potential Mediators of Reduced Mammary Tumor Growth by Chloroquine and the mTOR Inhibitor RAD001. Biochem Pharmacol (2012) 83(4):480–8. doi: 10.1371/journal.pone.0170298 22142888

[62] Albakr L Alqahtani FY Aleanizy FS Alomrani A Badran M Alhindas H. Improved Delivery of miR-1296 Loaded Cationic Nanoliposomes for Effective Suppression of Triple Negative Breast Cancer. Saudi Pharm J (2021) 29(5):446–55. doi: 10.1016/j.jsps.2021.04.007 PMC8180610 34135670

[63] Chen G He M Yin Y Yan T Cheng W Huang Z. miR-1296-5p Decreases ERBB2 Expression to Inhibit the Cell Proliferation in ERBB2-Positive Breast Cancer. Cancer Cell Int (2017) 17(1):95. doi: 10.1186/s12935-017-0466-y 29089858 PMC5655974

[64] Shan X Wen W Zhu D Yan T Cheng W Huang Z. miR 1296–5p Inhibits the Migration and Invasion of Gastric Cancer Cells by Repressing ERBB2 Expression. PLoS One (2017) 12(1). doi: 10.1093/carcin/bgu133 PMC5242522 28099468

[65] Huang L Liu D. Nonviral Vectors for Gene Therapy: Physical Methods and Medical Translation. Wagner E, editor. Elsevier Science (2015).

[66] Chen Y Gao D-Y Huang L. *In Vivo* Delivery of miRNAs for Cancer Therapy: Challenges and Strategies. Adv Drug Deliv Rev (2015) 81:128–41. doi: 10.18433/jpps31434 PMC5009470 24859533

[67] Humphries B Wang Z Oom AL. MicroRNA-200b Targets Protein Kinase Cα and Suppresses Triple-Negative Breast Cancer Metastasis. Carcinogenesis (2014) 35(10):2254–63. doi: 10.1515/jaots-2013-0205 PMC4178468 24925028

[68] D'Ippolito E Iorio MV. MicroRNAs and Triple Negative Breast Cancer. Int J Mol Sci (2013) 14(11):22202–20. doi: 10.1016/j.arabjc.2013.04.031 PMC3856060 24284394

[69] Nair AB Morsy MA Shinu P Kotta S Chandrasekaran M Tahir A. Advances of Non-Iron Metal Nanoparticles in Biomedicine. J Pharm Pharm Sci (2021) 24:41–61. 10.18433/jpps31434 33508214

[70] Umar K Haque MM Mir NA Muneer M Farooqi IH. Titanium Dioxide-Mediated Photocatalysed Mineralization of Two Selected Organic Pollutants in Aqueous Suspensions. J Adv Oxid Technol (2013) 16:252–60. doi: 10.1515/jaots-2013-0205

[71] Ramsurn H Ram B. Hydrogenation by Nanoparticle Catalysts. In: Gupta, in New and Future Developments in Catalysis (2013) (United States, Elsievier). pp.347–74.

[72] Hasan SA. Review on Nanoparticles: Their Synthesis and Types. Res J Recent Sci (2015) 4:1–3. doi: 10.1155/2017/5107485

[73] Kong T Zeng J Wang X Yang X Yang J McQuarrie S. Enhancement of Radiation Cytotoxicity in Breast-Cancer Cells by Localized Attachment of Gold Nanoparticles. Small (2008) 4(9):1537–43. doi: 10.1002/smll.200700794 18712753

[74] Andey T Sudhakar G Marepally S Patel A Banerjee R Singh M. Lipid Nanocarriers of a Lipid-Conjugated Estrogenic Derivative Inhibit Tumor Growth and Enhance Cisplatin Activity Against Triple-Negative Breast Cancer: Pharmacokinetic and Efficacy Evaluation. Mol Pharm (2015) 12(4):1105–20. doi: 10.1002/adhm.201300090 25661724

[75] Guo D Zhao Y Zhang Y Wang Q Huang Z Ding Q. The cellular uptake and cytotoxic effect of silver nanoparticles on chronic myeloid leukemia cells. J Biomed Nanotechnol (2014) 10(4):669–78. doi: 10.1166/jbn.2014.1625 24734519

[76] Fahrenholtz CD Swanner J Ramirez-Perez M Singh RN. Heterogeneous Responses of Ovarian Cancer Cells to Silver Nanoparticles as a Single Agent and in Combination With Cisplatin. J Nanomater (2017) 2017:5107485. doi: 10.1096/fba.2019-00021 30034459 PMC6052800

[77] Sriram MI Kanth S Kalishwaralal K Gurunathan S. Antitumor Activity of Silver Nanoparticles in Dalton's Lymphoma Ascites Tumor Model. Int J Nanomed. (2010) 5:753–62. doi: 10.2147/IJN.S11727 PMC2962271 21042421

[78] Sharma S Chockalingam S Sanpui P Chattopadhyay A Ghosh SS. Silver Nanoparticles Impregnated Alginate-Chitosan-Blended Nanocarrier Induces Apoptosis in Human Glioblastoma Cells. Adv Healthcare Mater (2014) 3(1):106–14. doi: 10.1016/j.ijpharm.2017.06.026 23852919

[79] Swanner J Fahrenholtz C Tenvooren I Bernish B Sears J Hooker A. Silver Nanoparticles Selectively Treat Triple-Negative Breast Cancer Cells Without Affecting non-Malignant Breast Epithelial Cells *In Vitro* and *In Vivo* . FASEB Bioadv (2019) 1(10):639–60. doi: 10.1016/j.ejpb.2017.07.003 PMC6996381 32123812

[80] Haque S Cook K Sahay G Sun C. RNA-Based Therapeutics: Current Developments in Targeted Molecular Therapy of Triple-Negative Breast Cancer. Pharmaceutics (2021) 13(10):1694. doi: 10.3390/pharmaceutics13101694 34683988 PMC8537780

[81] Viard M Koyfman AY Martins AN Kasprzak WK Panigaj M Desai R. Shapiro Multifunctional RNA Nanoparticles. Nano Lett (2014) 14(10):5662–71. doi: 10.1080/1061186X.2018.1523418 PMC4189619 25267559

[82] Shu D Li H Shu Yi Xiong G Carson WE3 Haque F. Systemic Delivery of Anti-miRNA for Suppression of Triple Negative Breast Cancer Utilizing RNA Nanotechnology. ACS Nano (2015) 9(10):9731–40. doi: 10.3109/10717544.2013.834412 PMC4723066 26387848

[83] Liu Li J Liu N Guo N Gao C Hao Y. *In Vitro* Studies of Phospholipid-Modified PAMAM-Simdr1 Complexes for the Reversal of Multidrug Resistance in Human Breast Cancer Cells. Int J Pharm (2017) 530:291–9. doi: 10.1016/j.ejpb.2013.07.002 28619457

[84] Chittasupho Anuchapreeda S Sarisuta N. CXCR4 Targeted Dendrimer for Anticancer Drug Delivery and Breast Cancer Cell Migration Inhibition. Eur J Pharm Biopharm. (2017) 119:310–21. doi: 10.1056/NEJMoa2028485 28694161

[85] Chen Y Zhang Y. Application of the CRISPR/Cas9 System to Drug Resistance in Breast Cancer. Adv Sci (2018) 5(6):1700964. doi: 10.1001/jamaoncol.2020.2965 PMC6010891 29938175

[86] De Sousa Cunha Nayra L Pryscilla T Alves R Mendes A. Development of Nanoparticulate Systems With Action in Breast and Ovarian Cancer: Nanotheragnostics, J. Drug Target (2018) 27:732–41. doi: 10.1016/j.hemonc.2014.11.003 30207742

[87] Acevedo-Morantes CY Acevedo-Morantes MT Suleiman-Rosado D Ramírez-Vick JE. Evaluation of the Cytotoxic Effect of Camptothecin Solid Lipid Nanoparticles on MCF7 Cells. Drug Delivery (2013) 20:338–48. doi: 10.1155/2012/385978 24024505

[88] Kaur T Slavcev R. Solid Lipid Nanoparticles: Tuneable Anti-Cancer Gene/Drug Delivery Systems. (2013), 53–73. doi: 10.1155/2012/385978

[89] Hadinoto K Sundaresan A Cheow WS. Lipid–polymer Hybrid Nanoparticles as a New Generation Therapeutic Delivery Platform: A Review. Eur J Pharm Biopharm. (2013) 85:427–43. doi: 10.1097/01.NAJ.0000660012.84038.48 23872180

[90] Zhang P Ling G Pan X. Novel Nanostructured Lipid-Dextran Sulfate Hybrid Carriers Overcome Tumor Multidrug Resistance of Mitoxantrone Hydrochloride. Nanomed Nanotechnol Biol Med (2012) 8:185–93. doi: 10.1093/annonc/mdy024 21704599

